# The prevalence of adjustment disorder and predisposing factors in infertile women

**DOI:** 10.1186/s40359-023-01193-4

**Published:** 2023-05-02

**Authors:** Shiva Shafierizi, Zahra Basirat, Fatemeh Nasiri-Amiri, Farzan Kheirkhah, Mohammad Chehrazi, Hajar Pasha, Mahbobeh Faramarzi

**Affiliations:** 1 Counselling in Midwifery, Student Research Committee, Babol University of Medical Sciences, Babol, Iran; 2 Department of Obstetrics and Gynecology, Infertility and Reproductive Health Research Center, Health Research Institute, Babol University of Medical Sciences, Babol, Iran; 3 Department of Reproductive Health, Infertility and Reproductive Health Research Center, Health Research Institute, Babol University of Medical Sciences, Babol, Iran; 4 Department of Psychiatry, Social Determinants of Health Research Center, Health Research Institute, Babol University of Medical Sciences, Babol, Iran; 5 Department of Biostatistics and Epidemiology, School of Public Health, Babol University of Medical Sciences, Babol, Iran; 6 Department of Reproductive Health, Social Determinants of Health Research Center, Health Research Institute, Babol University of Medical Sciences, Babol, Iran; 7 Department of Psychology, Infertility and Reproductive Health Research Center, Health Research Institute, Babol University of Medical Sciences, Babol, Iran

**Keywords:** Infertility, Adjustment disorder, Clinical presentation, Stress, Predisposing factors, COVID-19

## Abstract

**Background:**

Infertility is a stressful life event that increases the risk of developing mental disorders, particularly adjustment disorder (AD). Given the paucity of data on the prevalence of AD symptoms in infertility, the purpose of this study was to ascertain the prevalence, clinical presentation, and risk factors for AD symptoms in infertile women.

**Method:**

In a cross-sectional study, 386 infertile women completed questionnaires including the Adjustment Disorder New Module-20 (ADNM), the Fertility Problem Inventory (FPI), the Coronavirus Anxiety Scale (CAS), and the Primary Care Posttraumatic Stress Disorder (PC-PTSD-5) at an infertility center between September 2020 and January 2022.

**Result:**

The results indicated that 60.1% of infertile women exhibited AD symptoms (based on ADNM > 47.5). In terms of clinical presentation, impulsive behavior was more common. No significant relationship was observed between prevalence and women's age or duration of infertility. Infertility stress (β = 0.27, *p* < 0.001), coronavirus anxiety (β = 0.59, *p* = 0.13), and a history of unsuccessful assisted reproductive therapies (β = 2.72, *p* = 0.008) were several predisposing factors for AD symptoms in infertile women.

**Conclusions:**

The findings suggest that all infertile women be screened from the start of infertility treatment. Additionally, the study suggests that infertility specialists should focus on combining medical and psychological treatments for individuals predisposed to AD, particularly infertile women who exhibit impulsive behaviors.

## Introduction

Reproduction is an essential part of human life; thus, infertility and its treatment as a stressor may cause significant psychological and social harm [1]. According to estimates, approximately 10% to 12% of the world's population is affected by infertility [2]. Women are more likely to risk of negative effects of infertility than men such as feelings of guilt and isolation, social isolation, low self-esteem, decreased sexual satisfaction, and a lower quality of life [3–5]. Mental disorders are prevalent among 50% of infertile women [6, 7]. Most common emotional distresses are depression, anxiety, dysthymia, major depressive disorder (MDD), adjustment disorders (AD), and psychological stress [7–9]. Mental disorders have been linked to poor clinical outcomes with assisted reproductive technology (ART) [10, 11].

Infertile couples have significant adjustment problems. One-third of infertile people are at risk of emotional maladjustment before starting ARTs [12]. The prevalence of AD associated with infertility has been reported to range between 16 and 60% [7, 13]. AD refers to maladaptive behavioral and emotional responses to a specific external stressor within three months after the onset of the stressor (infertility). The symptoms (anxiety moods and depressed mood) do not continue for more than six months after termination of the stressor. Also, the symptoms must affect the individual’s daily activities, such as social or occupational functioning [14]. The primary distinction between AD and other stress-related illnesses is that AD is diagnosed following exposure to a significant life stressor that is not necessarily life-threatening (e.g., divorce, infertility, and bereavement) [15]. AD has been described in various clinical presentations as AD with depressed mood, anxiety, mixed anxiety and depressed mood, disturbance of conduct, mixed disturbance of emotions and conduct, and unspecified [16].

According to a more comprehensive definition, AD has six clinical presentations, including (1) Preoccupations: excessive worry, recurring thoughts, or rumination about the stressor or its consequences; (2) Failure to adapt: Difficulty coping with significant aspects of life, such as work, relationships, or recreational activities, as manifested by sleep disorders or concentration difficulties; (3) Avoidance: A cognitive or behavioral attempt to avoid distressing thoughts, feelings, or behaviors; (4) Symptoms of anxiety: worry; (5) Depression: sadness, unhappiness, and despair; (6) Impulsive behaviors: Actions taken without regard for the consequences [17–20].

Numerous factors influence the incidence, severity, and exacerbation of AD symptoms after a stressful event. Seventy studies were included in a systematic review to determine AD predictors. Female gender, younger age, unemployment, stress, illness, physical injury, lack of social support, and a history of mental health disorders were associated with an increased risk of developing AD symptoms [21]. There is a dearth of information regarding the risk factors for AD symptoms linked with infertility. Certain studies have identified the infertility process and the outcome of assisted reproductive technology as significant determinants of infertility incompatibility [22, 23]. Moura-Ramos et al. (2016) proposed that factors affecting emotional adjustment include the duration of pregnancy and the number and history of previous ARTs [22].

Few studies have examined the prevalence of AD symptoms and its clinical presentation in women experiencing infertility. Additionally, there is scant evidence of factors influencing AD symptoms in infertile women. Given the stressful nature of infertility and the potential for environmental stressors such as disease outbreaks to exacerbate psychological problems in this population, we investigated the prevalence of AD symptoms and its clinical presentation during the COVID-19 period. To our knowledge, this is the first study to investigate the prevalence, clinical presentation of AD, and predisposing factors in infertile women during the COVID-19 pandemic. A better understanding of the factors contributing to AD symptoms in infertile women during treatment can assist gynecologists/ healthcare providers in identifying which patients have more adjustment difficulties and require additional support.

The research hypotheses are as follows:

1. AD symptoms will be common in infertile women.
2. AD symptoms will be related to infertility stress.
3. AD symptoms will be associated to infertile women's demographic characteristics.
4. Some demographic characteristics of women and COVID-19 anxiety will be predicted the AD symptoms
5. Some demographic characteristics of women and COVID-19 anxiety will be predicted the infertility stress.

## Materials and methods

### Data collection

The present cross-sectional study was conducted at the Infertility and Health Reproductive Research Center of Babol University of Medical Sciences from September 2020 to January 2022. This study was performed during the third to the fifth waves of COVID-19 in Iran. The third wave started in November 2020, the fourth wave was in April 2021, and the fifth wave occurred in august 2021. generally, Iran being frequently among the countries with the highest morbidity and mortality, has faced numerous challenges [24]. Infertile women, who visited the Infertility Center, were invited to participate voluntarily during the study sampling period. The sample size was determined to be 386 patients based on pilot data obtained prior to the study (*p* = 0.49, α = 0.05, and d = 0.5).

The following criteria were used as the inclusion criteria: at least 18 years of age, a minimum of a fifth-grade education, not currently receiving psychotherapy, not pregnant, and willingness to participate in the study. The following volunteers were excluded from the study: report of mental retardation, self-reporting severe psychiatric disorders such as psychotic disorder, bipolar disorder, and substance abuse. As sever psychiatric disorders may lead to emotional and cognitive instability; therefore, participants who experienced psychiatric disorders at the time of the study were not appropriate eligible to answer the questionnaires. Also, women who had experienced stressful life event six months ago, except infertility (such as loss of first degree relative, presence of acute illness, family divorce, and loss of job) were not included the study. In order to control infertility as a possible cause of AD symptoms, patients who experienced other stressful life event in the last 6 months as confounding factors of AD symptoms arisen of the infertility were excluded in the study.

The midwives at the infertility center invited fertile women who visited the clinic to begin treatment and referred them to a room in the center during the sampling. A research team member (first author) gathered the obtained the information from patients’ medical records. The cause of infertility was based on the diagnosis by obstetricians. The duration of infertility was calculated from one year after unprotected coitus and trying to conceive for women younger than 35 and after six months of unprotected sex for women aged 35 years or older. Also, she conducted in-person interviews with all infertile women and reviewed the inclusion and exclusion criteria. The research project and objectives were explained if an individual was eligible for inclusion. Patients who agreed to participate in the study were instructed to complete the questionnaires. Participants were given the option of filling out paper questionnaires inside the clinic or receiving a questionnaire link (via the DigiSurvey® platform) via WhatsApp® or Telegram® to complete at home, within a week or less. A total of 460 individuals were invited to participate in the study, of which 60 (43 were not satisfied to participate in the study, 5 having attended psychotherapy sessions, 4 were illiterate, 8 reported severe psychological disorders) were deemed ineligible. A total of 400 infertile women were enrolled in the study and completed questionnaires. Among them, 151 responded to online questionnaires, while 235 responded to paper questionnaires. Fourteen questionnaires were discarded due to inaccuracies in item completion, leaving 386 for analysis.

### Measures

#### Adjustment Disorder-New Module 20 (ADNM-20)

This questionnaire is a self-report instrument that assesses symptoms on a four-point scale ranging from 1 (never) to 4 (often) [25]. As our aim was to select the participants whose adjustment disorder arisen from infertility, we mentioned infertility as the stressor for each item of the ADNM-20 questionnaire. Preoccupation, failure to adapt, avoidance, depressive mood, anxiety, and impulsive disturbance are sub-components of this questionnaire. The sub-components titled preoccupation and failure to adapt assess the primary symptoms. The total score is calculated by adding the scores of all 20 items. The total score ranges from 20 to 80. The questionnaire has a cut-off of 47.5 for AD symptoms [26]. The study employed a validated Persian version of the questionnaire [27]. The Cronbach's alpha of the questionnaire was 0.87, which indicates good reliability of the whole questionnaire. The cut-off score of 47.5 was not applied to the Persian version.

#### Fertility Problem Inventory (FPI)

This questionnaire was developed by Newton (1999) [28]. This scale is used to assess stress and infertility. The FPI consists of 46 questions and five sub-components organized around five themes: sexual concern, social concern, relationship concern, parental need, and rejection of a childfree lifestyle. A 6-point Likert scale is employed from 1 (strongly disagree) to 6 (strongly agree). The total score range is 46–276, and higher scores indicate higher stress levels. A validated Persian version was used in this study [29]. Cronbach's alpha for all sub-scales was more than 0.7. Also, the overall integrity using McDonald's Omega was 0.92.

#### Coronavirus Anxiety Scale (CAS)

This questionnaire was developed by Lee (2020) [30]. COVID-19 anxiety is assessed using a five-item scale. CAS measures emotional, cognitive, behavioral, and physiological aspects related to coronavirus anxiety for the last two weeks. Each item is graded on a 5-point scale (0 being never to 4 being almost every day). A validated Persian version was used in this study [31]. Internal consistency using McDonald's Omega was 0.75.

#### Primary Care Posttraumatic Stress Disorder (PC-PTSD-5)

This questionnaire is a five-question self-report screening, which has been developed for use in primary care settings. Scores are assigned on a scale of 0 to 1 (0 equals no; 1 equals yes) and assessed stress related symptoms. The total score is calculated by adding the answers to five questions [32]. A cut-off score of 3 is recommended for PTSD symptoms [32]. A validated Persian version was used in this study [33]. The computed Cronbach's alpha for the Persian version of PC-PTSD was 0.7.

### Statistical analysis

The mean demographic variables were compared between participants presenting symptom of AD and normal groups using the t-test (for variables with a normal distribution) and the Mann–Whitney test (for variables with abnormal distribution). The kolmogorov–Smirnov test was used to assess normal distribution. Additionally, the t-test and ANOVA were used to compare the means of psychological profiles based on demographic characteristics. We used also chi-square test to compare different between categorical variables of AD and non-AD group. Finally, we used multivariate linear regression models with age, job, education level, medical history, history of substance abuse, marriage duration, duration of infertility, history of assisted reproductive technology failure, the total score of coronavirus anxiety, the total score of infertility stress, and total score of coronavirus anxiety [9, 22, 34, 36, 37, 40–53] as independent variables along with AD symptoms and infertility stress as dependent variables in two separate models. SPSS software (v. 18) was used to analyze data. *p*-value < 0.05 was considered the significance level.

### Ethical approval

The present study was approved by the ethics committee of Babol University of Medical Sciences (MUBABOL.HRI.REC.1399.105). Prior to participation in the study, all participants provided written informed consent. Additionally, the participants' anonymity and confidentiality of their information were guaranteed.

## Results

The demographic characteristics of the research population are summarized in Table 1. The participants' mean age was 32.4 ± 5.9. Most participants were homemakers (74.3) and urban dwellers (58.2). In terms of causes, the prevalence of factors was as follows: female factor (18.6), male factor (28.2), male and female factor (25), and unknown factor (28.2). The mean duration of infertility (years) was 5.1 ± 4.1.

**Table 1:** Prevalence of adjustment disorder symptoms regarding to characteristics of the study population

Variables	Total (sample) N = 386	With AD symptoms ^‡^ group (N = 232)	Without AD symptoms group (N = 154)	*p*-value
Age, Mean ± SD	32.4 ± 5.9	31.9 ± 5.8	33.0 ± 6.1	0.092*
Education, N (%)				
Under diploma	63 (17.5)	45 (71.4)	18 (28.6)	
Diploma	119 (33.1)	78 (65.5)	41 (34.5)	0.044
University	177 (49.3)	98 (55.4)	79 (44.6)	
Employment status				
Employed	283 (74.3)	178 (62.9)	105 (37.1)	0.026
Unemployed	98 (25.7)	50 (51)	48 (49)	
Place of residence				
Urban	223 (58.2)	123 (55.2)	100 (44.8)	0.014
Rural	160 (41.8)	107 (66.9)	53 (33.1)	
Medical illness				
Yes	117 (31.9)	78 (66.7)	39 (33.3)	0.046
No	250 (68.1)	142 (56.8)	108 (43.2)	
Cause of infertility				
Woman	70 (18.6)	38 (54.3)	32 (45.7)	
Man	106 (28.2)	52 (49.1)	54 (50.9)	0.012
Both	94 (25)	66 (70.2)	28 (29.8)	
Unknown	106 (28.2)	68 (64.2)	38 (35.8)	
Failure of previous				
Treatment ART	185 (48.6)	126 (64.3)	70 (35.7)	0.043
Yes	196 (51.4)	102 (55.1)	83 (44.9)	
No				
Infertility duration, median (IQR)	5 (3.0, 8.0)	5 (3.0, 8.0)	5 (2.75, 8.0)	0.220**

Adjustment disorder^‡^: ADNM scores > 47.5, N; Without AD: ADNM scores ≤ 47.5, Total adjustment disorder: 20–80

*t-test, ** Mann–Whitney Test, Qualitative variables: χ^2^

*p* < 0.05 considered significant

AD symptoms were prevalent in 60.1% of infertile women based on ADNM > 47.5. The disorder was higher in women who had previously failed assisted reproductive therapy than women who had never used assisted reproductive techniques (64.3% vs. 55.1%, *p* = 0.043). Additionally, rural women had a higher prevalence of AD symptoms than urban women (66.9% vs. 55.2%, *p* = 0.014). AD symptoms were more common among non-university educated individuals than academics (67.8% vs. 55.4%, *P* = 0.044). The incidence was higher in participants with a history of medical disease than in those without (66.7% vs. 56.8%, *p* = 0.046). Concerning prevalence based on infertility factor causes, the highest level of AD symptoms was observed in women who had a common cause of female/male factor (70.2%), followed by an unknown factor (64.2%), male factor (49.1%), and female factor (54.3%) (*p* = 0.012). Unemployed women had a higher rate of AD symptoms than employed women (62.9% vs. 51%, *p* = 0.026). The failure of assisted reproductive therapies, comorbidities, lower education, infertility due to a shared female/male factor, and unemployment contributed to the rise in AD symptoms prevalence. However, AD symptoms was not significantly associated with female infertility duration or age.

The relationship between mean AD scores and subcomponents and demographic characteristics of infertile women is shown in Table 2. AD symptoms and none of its subcomponents were found to have a significant relationship with age. The subcomponents of failure to adapt, depressive mood, anxiety and total scores of AD were significantly higher in the under diploma education group than in the university group. Total AD (*p* = 0.029) and failure to adapt (*p* = 0.005) scores were higher in infertile women living in rural areas than in urban areas. Except for the failure to adapt and avoidance, the mean total scores of AD and its three subcomponents were higher among participants who experienced female/male infertility than those who experienced male factor infertility. Furthermore, the anxiety sub-component was higher in the infertility group with unknown factors than in the male factor group (*p* = 0.004). The total mean scores of the AD (*p* = 0.048), impulsive disturbance (*p* = 0.048), and preoccupation (*p* = 0.008) subcomponents were significantly different between those who had previously received assisted reproductive treatment and those who had not received ART.

**Table 2:** Comparison of mean (SD) scores of the adjustment disorder new module (ADNM) and subscales regarding demographic characteristic of infertile woman

Variables	Preoccupation	Failure to adapt	Avoidance	Depressive mood	Anxiety	Impulsive disturbance	Total ADNM
	Mean ± SD	Mean ± SD	Mean ± SD	Mean ± SD	Mean ± SD	Mean ± SD	Mean ± SD
Age							
< 35	10.6 ± 3.1	8.7 ± 3.4	10.5 ± 2.8	7.9 ± 2.8	5.2 ± 1.7	8.6 ± 2.8	51.7 ± 12.8
≥ 35	10.3 ± 3.3	8.2 ± 3.6	10.1 ± 2.7	7.6 ± 2.3	4.9 ± 1.8	8.0 ± 2.9	
*p*-value	0.309	0.246	0.23	0.135	0.116	0.74	49.3 ± 14.0
							0.99
Education							
Under diploma	11.3 ± 2.9	10.0 ± 3.6^ab^	10.3 ± 2.8	8.4 ± 2.1^b^	5.6 ± 1.7^b^	9.1 ± 2.7	55.0 ± 13.1^b^
Diploma	10.5 ± 3.3	8.5 ± 3.3	10.5 ± 2.8	8.0 ± 2.0	5.0 ± 1.7	8.5 ± 2.9	51.3 ± 13.0
University	10.3 ± 3.1	8.1 ± 3.4	10.5 ± 2.7	7.5 ± 2.3	4.9 ± 1.7	8.2 ± 2.8	49.8 ± 13.5
*p*-value	0.107	0.001	0.9	0.015	0.023	0.075	0.029
Duration of infertility							
< 5	10.4 ± 3.0	8.2 ± 3.3	10.6 ± 2.7	7.7 ± 2.2	5.0 ± 1.7	8.3 ± 2.8	50.6 ± 12.9
5–9	10.9 ± 3.3	8.9 ± 3.5	10.5 ± 2.9	8.0 ± 2.1	5.2 ± 1.8	8.7 ± 2.8	52.5 ± 13.6
≥ 10	10.6 ± 3.1	9.4 ± 3.8	10.1 ± 2.8	7.8 ± 2.2	4.9 ± 1.7	8.8 ± 2.7	51.8 ± 13.5
*p*-value	0.513	0.064	0.396	0.481	0.39	0.289	0.456
Place of residence							
Urban	10.2 ± 3.2	8.0 ± 3.4	10.4 ± 2.9	7.6 ± 2.2	4.9 ± 1.8	8.1 ± 2.9	49.5 ± 13.6
Rural	10.8 ± 3.2	9.1 ± 3.5	10.6 ± 2.7	8.0 ± 2.1	5.2 ± 1.7	8.6 ± 2.7	52.6 ± 12.7
*p*-value	0.121	0.005	0.479	0.98	0.39	0.83	0.029
Cause of infertility							
Man	9.7 ± 3.2^d^	7.9 ± 3.4	10.1 ± 2.9	7.3 ± 2.3^d^	4.5 ± 1.8^de^	7.6 ± 2.9^d^	47.4 ± 13.1^d^
Woman	10.4 ± 2.9	8.2 ± 3.4	10.4 ± 2.9	7.4 ± 2.2	5.2 ± 1.8	8.2 ± 2.7	49.9 ± 13.0
Both	11.1 ± 3.2	8.9 ± 3.3	10.4 ± 2.6	8.2 ± 1.8	5.3 ± 1.4	9.2 ± 2.4	53.5 ± 12.2
Unknown	10.5 ± 3.3	8.7 ± 3.7	10.8 ± 2.7	8.1 ± 2.2	5.2 ± 1.8	8.4 ± 3.0	52.0 ± 14.0
*p*-value	0.022	0.141	0.283	0.007	0.004	0.001	0.007
Failure of treatment							
No	10.0 ± 3.2	8.2 ± 3.5	10.3 ± 2.8	7.7 ± 2.1	4.9 ± 1.7	8.0 ± 3.0	49.3 ± 13.3
Yes	10.9 ± 3.2	8.7 ± 3.4	10.6 ± 2.7	7.8 ± 2.2	5.1 ± 1.8	8.6 ± 2.7	52.0 ± 13.2
*p*-value	0.008	0.131	0.3	0.506	0.193	0.048	0.048

Range of scores: Preoccupation: 4–16, Failure to adapt: 4–16, Avoidance: 4–16, Depressive: 3–12, Anxiety: 2–8, Impulsive disturbance: 3–12, ADNM adjustment disorder new module: 20–80

a: significant difference between under diploma and diploma

b: significant difference between under diploma and university

d: significant difference between men and both

e: significant difference between men and unknown

The mean scores of the six types of AD subgroups were compared in terms of clinical presentation on a scale of 1 to 4. The results indicated that the mean (M ± SD) of clinical variables was as follows: Impulsive disturbance (0.5 ± 3.3) had the highest mean, and mean scores of anxiety (3.0 ± 0.6), depressive mood (3.0 ± 0.5), and preoccupation (0.5 ± 3.0) were very close, whereas the mean scores of avoidance (2.8 ± 0.5), and failure to adapt (2.6 ± 0.6) were the lowest.

The relationship between infertility stress and the demographic characteristics of infertile women is reported in Table 3. The findings indicated that neither the total score of infertility stress nor its sub-components, except for rejection of a childfree lifestyle (*p* = 0.005), had a significant relationship with age. Total infertility stress scores and all of the subcomponents (except rejection of childfree lifestyle) were significantly higher in participants with under diploma education levels than individuals with academic levels. Need for parenthood (*p* = 0.024) and rejection of childfree lifestyle (*p* = 0.04) scores were higher in infertile women experiencing 5–9 years of infertility compared to those who experienced infertility more than ten years. Except for the need for parenthood and rejection of a childfree lifestyle, the total scores of infertility stress and its subcomponents were higher in rural women than in urban women (sexual concern (*p* = 0.008), social concern (*p* = 0.009), and relationship concern (*p* < 0.001)). Furthermore, women who experienced infertility due to a female/male factor had higher total scores for infertility stress (*p* = 0.002), sexual concern (*p* = 0.001), relationship concern (*p* = 0.012), and rejection of a childfree lifestyle (*p* = 0.003) than those who experienced infertility due to male and female factors. However, there was no significant relationship between infertility stress and assisted reproductive therapy failure history.

**Table 3:** Comparison of mean (SD) scores of the infertility problem inventory (FPI) and subscales regarding demographic characteristic of infertile woman

Variables	Sexual concern	Social concern	Relationship concern	Need for parenthood	Rejection of childfree lifestyle	Total FPI
	Mean ± SD	Mean ± SD	Mean ± SD	Mean ± SD	Mean ± SD	Mean ± SD
Age						
< 35	21.5 ± 7.5	26.8 ± 8.5	26.1 ± 9.1	41.5 ± 9.0	30.9 ± 7.5	147.0 ± 31.0
≥ 35	22.4 ± 7.7	26.3 ± 9.5	26.3 ± 9.2	39.8 ± 8.6	28.6 ± 7.4	143.6 ± 33.3
*p*-value	0.311	0.616	0.806	0.079	0.005	0.326
Education						
Under diploma	25.1 ± 8.3^b^	30.5 ± 8.3^ab^	29.4 ± 9.0^b^	43.3 ± 7.9^b^	31.4 ± 7.9	159.8 ± 32.5^b^
Diploma	22.8 ± 7.5^c^	26.9 ± 8.9	27.3 ± 8.6^c^	42.0 ± 8.9	30.92 ± 6.9	150.2 ± 28.6^c^
University	20.3 ± 6.9	25.4 ± 8.7	24.6 ± 9.2	39.8 ± 9.0	29.1 ± 7.6	139.3 ± 31.7
*p*-value	< 0.001	< 0.001	0.001	0.011	0.051	< 0.001
Duration of infertility						
< 5	21.3 ± 7.0	26.4 ± 8.2	25.4 ± 8.8	40.4 ± 9.1	30.1 ± 7.7	143.8 ± 30.45
5–9	23.2 ± 8.2	27.3 ± 9.4	26.9 ± 9.7	42.5 ± 9.0^d^	31.0 ± 7.6^d^	151.1 ± 33.9
≥ 10	22.1 ± 7.7	26.3 ± 9.9	28.0 ± 9.2	38.8 ± 8.1	27.9 ± 6.9	143.3 ± 33.3
*p*-value	0.094	0.676	0.133	0.024	0.04	0.123
Place of residence						
Urban	20.9 ± 7.3	25.5 ± 8.6	24.4 ± 8.7	40.3 ± 9.1	29.4 ± 7.7	140.7 ± 31.2
Rural	23.0 ± 7.7	27.9 ± 9.2	28.1 ± 9.3	41.6 ± 8.8	30.7 ± 7.6	151.5 ± 33.0
*p*-value	0.008	0.009	< 0.001	0.19	0.091	0.001
Cause of infertility						
Man	20.2 ± 6.9^e^	25.1 ± 8.2	24.6 ± 9.5^e^	40.1 ± 8.9	28.4 ± 7.4^e^	138.63 ± 32.3^e^
Woman	20.1 ± 6.8^f^	26.0 ± 9.3	25.2 ± 9.3	41.7 ± 8.1	28.8 ± 7.6^f^	140.0 ± 30.4^f^
Both	23.8 ± 7.8	28.3 ± 8.6	28.6 ± 9.1	41.5 ± 8.1	32.2 ± 7.7	154.8 ± 31.8
Unknown	22.8 ± 7.5	26.7 ± 9.4	25.5 ± 8.4	40.8 ± 8.9	30.0 ± 7.6	146.7 ± 31.7
*p*-value	0.001	0.072	0.012	0.383	0.003	0.002
Failure of treatment						
No	21.5 ± 7.2	26.5 ± 8.3	25.9 ± 9.2	40.3 ± 9.1	30.1 ± 7.7	31.4 ± 2.3
Yes	21.8 ± 7.7	26.5 ± 9.4	25.9 ± 9.1	41.1 ± 8.7	29.7 ± 7.5	32.8 ± 2.3
*p*-value	0.666	0.941	0.932	0.394	0.63	0.821

Range of scores: Sexual concern: 8–48, Social concern: 10–60, Relationship concern: 10–60, Need for parenthood: 10–60, Rejection of childfree lifestyle: 8–48, FPI Fertility problem inventory: 46–276

a: significant difference between under diploma and diploma

b: significant difference between under diploma and university

c: significant difference between diploma and university

d: significant difference between 5–9 and ≥ 10

e: significant difference between men and both

f: significant difference between women and both

For determine the predisposing factors associated with AD symptoms, we used the multivariate linear regression analysis (Table 4). In this model, the total AD score was used as the dependent variable, while the total score of infertility stress, prior failure history in assisted reproductive therapy, coronavirus anxiety, job, education level, age, duration of infertility, disease history, smoking history, and duration of marriage were used as independent variables. The results showed that infertility stress (β = 0.27, *p* < 0.001) and coronavirus anxiety (β = 0.59, *p* = 0.13) were at risk of higher scores of AD. Also, women with a history of infertility treatment failure were at risk of higher score of AD than those without (β = 2.72, *p* = 0.008).

**Table 4:** Results of multivariate linear regression analysis for predisposing factors of adjustment disorder symptoms

Variables	Regression coefficients	95% Confidence interval	*p*-value
Infertility stress	0.27	0.24, 0.30	< 0.001
Failure of previous treatment			
No	Ref	0.71, 4.73	0.008
Yes	2.72		
Coronavirus anxiety	0.59	0.12, 1.05	0.013
R^2^ (adjusted R^2^)	0.462 (0.450)		

Ref: reference, Coronavirus anxiety scale: 0–20, infertility stress: Fertility problem inventory: 46–276

The results of multivariate linear regression analysis were used to for determining predisposing factors of infertility stress (Table 5). In this model, the total infertility stress score was considered as the dependent variable and previous failures in assisted reproductive therapy, coronavirus anxiety, job, education level, age, duration of infertility, disease history, smoking history, and duration of the marriage as the independent variables. Employed women reported less infertility stress than unemployed (β = −10.37, *p* = 0.005). Participants with a university education reported less stress related to infertility than those with less than a high school diploma (β = −16.46, *p* = 0.001). Women whose infertility was caused by a common female/male factor experienced increased infertility stress than women whose infertility was solely due to a female factor (β = 16.66, *p* = 0.002). Moreover, coronavirus anxiety was significantly risk factor of infertility stress (β = 1.70, *p* = 0.47).

**Table 5:** Results of multivariate linear regression analysis for predisposing factors of infertility stress

variables	Regression coefficients	95% Confidence interval	*p-*value
Job			
Employed	−10.37	−17.66, −3.07	0.005
Unemployed	Ref		
Education			
Under diploma	Ref		
Diploma	−6.37	−16.43, 3.69	0.214
University	−16.46	−26.27, −6.65	0.001
Duration of infertility			
< 5	Ref		
5–9	4.97	−2.57, 12.51	0.195
≥ 10	−9.06	−19.27, 1.15	0.082
Cause of infertility			
Woman	Ref		
Man	2.74	−7.22, 12.71	0.588
Both	16.66	6.42, 26.91	0.002
Unknown	8.94	−0.71, 18.59	0.069
Coronavirus anxiety	1.70	0.021, 3.37	0.047
R^2^ (adjusted R^2^)	0.492 (0.472)		

Ref: reference, Coronavirus anxiety scale: 0–20

## Discussion

The prevalence of AD symptoms was high in infertile women (60.1% based on ADNM > 47.5). Additionally, the most common clinical presentation of AD symptoms in infertile women was impulsive behavior. Other studies of the general population and adults found that the most common manifestation of AD symptoms were mixed anxiety and depressed mood [34], depression symptoms [35], and anxiety symptoms [36]. Studies on the prevalence of AD symptoms in infertile women reported a mixed presentation of anxiety and depression symptoms [7, 9, 37, 38]. The clinical presentation of this study may differ from that of other studies due to differences in the tools used to evaluate the symptoms of AD, different diagnostic methods (diagnostic interview/questionnaire), and sociocultural differences among the research population.

In one study, AD symptoms prevalence in infertile men and women was reported to be similar to the current study (59.6%) [38]. Patel et al. (2020) used the ICD-10 Classification of Mental and Behavioral Disorders to study 300 infertile women and found that the prevalence of AD in infertile women was significantly lower than in our study (16% of mixed AD) [7].

According to a previous study using similar tools and cut-offs (ADNM > 47.5), 61.3% of the general population experienced AD as a result of quarantine issues and the prevalence of COVID-19 [39]. This finding could result from various tools and reports of symptoms of AD patients experiencing both anxiety and depressive mood [7]. The present study included women with primary and secondary infertility and those who had repeated treatment cycle failures, whereas the study mentioned previously included women who had primary infertility prior to initiating their treatment cycle.

According to this study, the influential factors increasing the prevalence of AD symptoms in infertile women were a failure of assisted reproductive therapies, comorbidity, lower education, infertility caused by common female/male factors, and unemployment. Consistent with these findings, Yaseen (2017) studied outpatient psychiatric clinic patients and identified the most significant risk factors for AD as low education, younger age, and urban vs. rural areas [34].

In contrast to our findings, a previous study found that AD symptoms was more prevalent in women with female infertility factor than in women with male infertility [40]. Another study discovered that AD symptoms was more common among infertile women who began assisted reproductive techniques than among women who did not use assisted reproductive techniques [37].

Surprisingly, this study found no correlation between AD symptoms and infertility stress and age or duration of infertility. In contrast to these findings, most previous research indicated that the prevalence of infertility-related psychiatric disorders was directly related to the patient's age and duration of infertility [41–44]. However, only a few studies corroborated our findings. A study discovered that infertile women's treatment duration did not affect their psychiatric morbidity [9]. Another study of 406 infertile women and men found no significant association between age or duration of infertility and a psychiatric disorder [45]. According to Sbaragli et al. (2008), participants who experienced infertility for two years or more were more likely to be diagnosed with AD symptoms than those who experienced infertility for less than two years [40]. In another study, advanced age was identified as a risk factor for AD symptoms [46].

The findings from the study of factors affecting infertility stress in women indicated that women's jobs and educational levels were protective against infertility stress. Predisposing factors for infertility included common female/male factors and coronavirus anxiety. In line with these findings, Lei et al. (2021) identified low education levels and rural areas as risk factors for infertile men and women experiencing infertility stress [47]. Another study on infertile women found that the duration of infertility and the relative importance of unknown causes of infertility to other causes of infertility were factors influencing infertile women's stress, but that stress had no significant relationship with education level or age [48].

Several studies were identified that differed from the current study. Stress was not associated with the duration of infertility in a study of 435 infertile women [49]. Zurlo et al. observed that infertile individuals with a higher level of education expressed more social concern and had a lower need for parenthood and rejection of a childfree lifestyle. In male and female patients, the female/male factor was associated with higher levels of all subscales and overall scores [50]. Various studies have confirmed COVID-19's beneficial effect on stress and distress in infertile women [49, 51, 52]. COVID-19 had significant effects, including delaying or terminating infertility treatment and increasing psychological distress following quarantine.

We found that infertility stress and a history of unsuccessful assisted reproductive therapies increased the odds ratio of infertile women developing AD symptoms. A literature review revealed no link between AD symptoms and history of assisted reproductive therapy failure or infertility stress. Moura-Ramos et al. (2016) observed that the duration of infertility and the number of failed assisted reproductive therapies did not affect infertile couples' emotional adjustment [22]. Several prior studies established a link between AD and perceived stress [36, 53].

The current study had several limitations that should be considered when generalizing the findings. First, the study was conducted throughout the two-year duration of the COVID-19 pandemic. Since coronavirus anxiety was a factor in the prevalence of AD symptoms and infertility stress, it is prudent to extrapolate this finding to periods other than COVID-19. Second, coronavirus anxiety, particularly during the epidemic's peak, played a significant role in some clients opting out of fertility treatments. Additionally, infertility clinics were prohibited from admitting clients to begin treatment during the COVID-19 peak. These factors may contribute to a population selection bias that does not accurately reflect the infertile population. Third, this study used a questionnaire to determine symptom of AD. Future research should incorporate a clinical diagnostic interview based on the DSM-5 and a questionnaire. Because the study was cross-sectional, its cause-and-effect relationships are suspect. Future research should use a prospective cohort design to examine the prevalence of AD symptoms and effective factors in infertile couples from the start of their infertility journey, mainly from commencing assisted reproductive technologies.

Despite the limitations mentioned above, the current study possessed considerable strength. It was the first study to our knowledge to examine the prevalence of AD, its clinical presentation, and its determinants in the field of infertility. Moreover, the large sample size was strength of the current study during the period of acute social stress associated with the COVID-19 epidemic.

These findings have clinical implications for infertility settings. The finding that AD symptoms is identical in young and old infertile women and is also unrelated to the duration of infertility expands the horizons of infertility specialists by indicating that infertile women who are new to infertility are just as likely to be exposed to infertility AD symptoms as older women or those with a longer duration of infertility. Thus, all infertile women should be screened for mental disorders, particularly AD symptoms, as soon as they enter infertility clinics and concurrently with requesting infertility treatment.

Furthermore, the findings indicated which women were predisposed to AD symptoms and thus required additional psychological care and support. Individuals who have failed infertility treatments should receive special attention from infertility specialists, as their odds ratios for developing AD symptoms increase with infertility. Because AD impulsive behavior is the most frequently encountered clinical presentation in infertile patients, infertility specialists and caregivers should be aware that symptoms of this disease may manifest in the physician–patient relationship, including sudden aggression, abrupt withdrawal from treatment, and immature behaviors. Given that infertility stress is a significant factor influencing the prevalence of AD symptoms and that the cause of infertility was associated with common female/male factors in unemployed and low-educated individuals, these findings suggest that gynecologists and caregivers of infertile women should place individuals with a higher proclivity for AD symptoms under increased supervision and care.

In conclusion, AD symptom was found to be prevalent in more than 60% of infertile women, with impulsive behavior being the most common presentation. Additionally, the prevalence of infertility was as high in young women and those newly diagnosed with infertility problems as in older women with a long history of infertility. Stress associated with infertility, a history of unsuccessful assisted reproductive therapy, and coronavirus anxiety increased the odds ratio of infertile women developing AD symptoms during the COVID-19 epidemic. These findings suggest that gynecologists and other healthcare providers in infertility clinics should begin AD symptoms screening for all women (young and old) before starting assisted reproductive treatments. Furthermore, they should focus on those at risk of developing AD, those at high risk of infertility, such as unemployed women and those with low education levels, and infertility caused by female/male factors. These findings suggest that assisted reproductive therapies should be combined with psychological support for individuals at risk of developing AD, particularly infertile women with impulsive behaviors.

## Data Availability

The data that support the findings of this study are available from the corresponding author upon reasonable request.

## Abbreviations

AD: Adjustment disorder
MDD: Major depressive disorder
ART: Assisted reproductive technology
ADNM-20: Adjustment Disorder-New Module 20
FPI: Fertility Problem Inventory
CAS: Coronavirus Anxiety Scale
PC-PTSD-5: Primary Care Posttraumatic Stress Disorder
ICD-10: International classification of diseases 10th edition
